# Zirconium nanoparticles prepared by the reduction of zirconium oxide using the RAPET method

**DOI:** 10.3762/bjnano.2.23

**Published:** 2011-04-06

**Authors:** Michal Eshed, Swati Pol, Aharon Gedanken, Mahalingam Balasubramanian

**Affiliations:** 1 Department of Chemistry, Kanbar Laboratory for Nanomaterials, Nanotechnology Research Center, Institute of Nanotechnology and Advanced Materials, Bar-Ilan University, Ramat-Gan 52900, Israel; 2 Advanced Photon Source, Argonne National Laboratory, Argonne IL 60439, USA

**Keywords:** Let-Lok^®^, nanoparticles, RAPET, reduction, zirconium

## Abstract

The aim of the current work is the synthesis and characterization of metallic Zr nanoparticles. The preparation is carried out by using the RAPET method (Reaction under Autogenic Pressure at Elevated Temperatures) developed in our lab. The RAPET reaction of commercial ZrO_2_ with Mg powder was carried out in a closed stainless steel cell, at 750 °C. On completion of the reaction, the additionally formed MgO is removed by treatment with acid. The characterization of the product was performed by XRD, X-ray absorption spectroscopy, SEM, TEM and elemental analysis. The XRD pattern reveals that the product is composed of pure metallic zirconium, without any traces of the MgO by-product.

## Introduction

Zirconium is a strong transition metal that resembles titanium. Because of its strong resistance to corrosion [1], it is used as an alloying agent in materials that are exposed to corrosive agents such as surgical appliances, explosive primers, vacuum tube getters and filaments. Since it has a very negative reduction potential (−1.55 V), it is never found as the native metal. It is obtained mainly from the mineral zircon, which can be purified with chlorine [2]. Zirconium metal is also used for making zirconium inorganic and organic compounds. Many examples of the synthesis of zirconium complexes for catalytic applications are described in the literature [3–4]. A very important example for the application of metallic Zr is the use of Zr nanoparticles (NP) as catalysts for growing TWCNTs (two or three graphene layer tubing) [5]. The presence of the zirconium as a catalyst ensures an effective method for the synthesis of high purity and good quality CNTs.

The best known process for the production of metallic zirconium is the Kroll process [6]. In this reaction zirconium is produced by the reduction of zirconium tetrachloride with an active metal such as magnesium at 800–900 °C. Elsewhere in the literature, the preparation of metallic Zr nanoparticles by ultrafast laser ablation of a zirconium rod in isopropyl alcohol has been described [7]. This process produces a colloidal solution of zirconium nanoparticles. Moreover, it was shown that the size distribution of nanoparticles can be greatly reduced by employing femtosecond laser pulses for ablation. A plasma induced cathodic discharge electrolysis under Ar gas in molten salt has also been used for fabricating 50 nm metallic zirconium nanoparticles [8]. Implants are frequently made of zirconium as well as from titanium [9]. In the powder form, zirconium is highly flammable, and consequently has military applications [10], such as in the production of explosive materials for munitions.

In this paper we report the successful synthesis of metallic Zr nanoparticles. The reaction is carried out by reacting zirconia with magnesium at 750 °C in a closed Let-Lok^®^ cell. We have named this process RAPET (Reaction under Autogenic Pressure at Elevated Temperatures), and is described elsewhere in detail [11]. In general, a stainless steel cell is filled with a single reactant (or a combination of reactants) and heated at an appropriate temperature under its autogenic pressure. The pressure is generated by the thermal dissociation of reactants and the Let-Lok^®^ union serves as an autoclave with a relief valve at 160 atm. The advantages of using the Let-Lok^®^ union is that it can be heated to 1000 °C. There are no commercial autoclaves available for temperatures higher than 650 °C at high pressure.

## Results and Discussion

**Figure 1:**
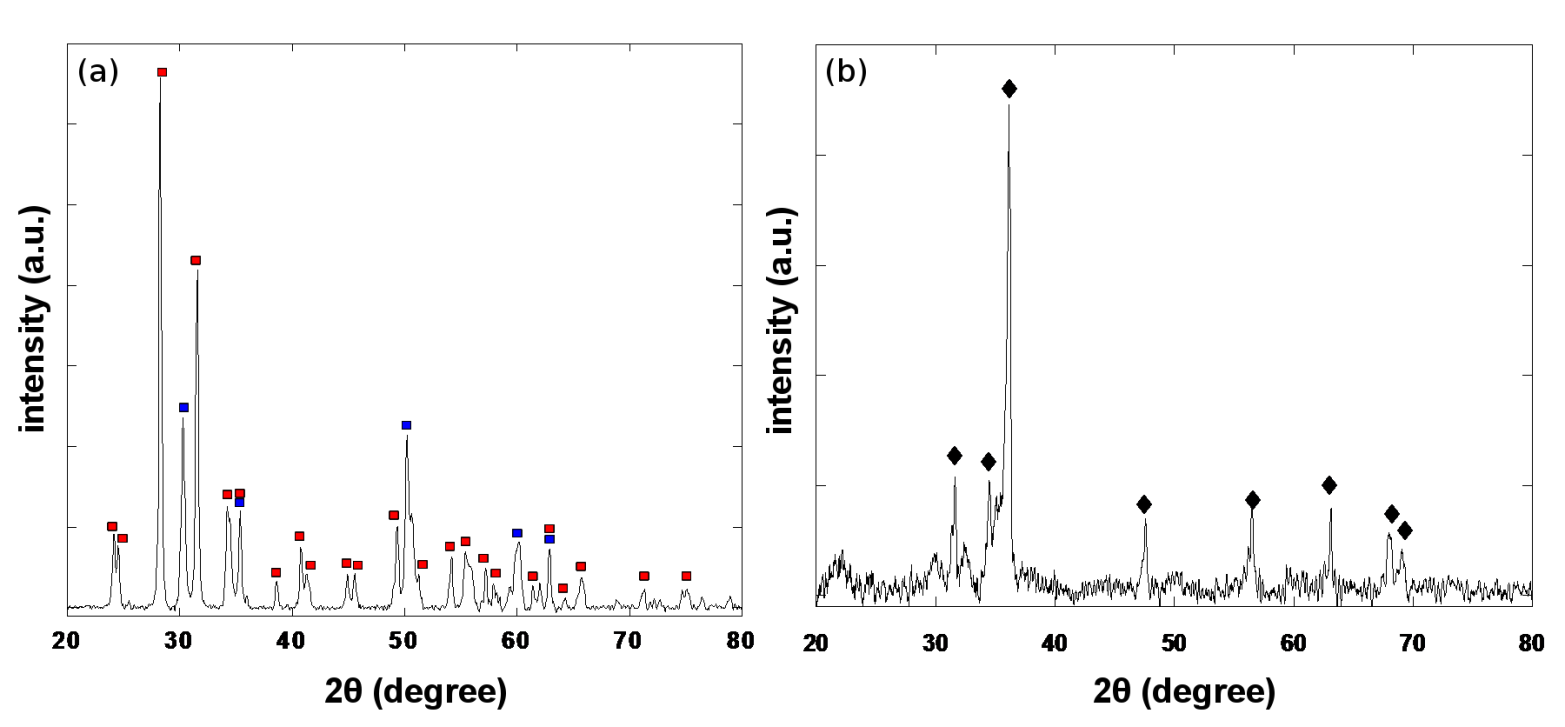
(a) XRD pattern of ZrO_2_ precursor, (b) XRD pattern of the synthesized zirconium product after acid treatment.

Both the precursor, zirconium dioxide, and the product were characterized by PXRD (Powder X-Ray Diffraction). Figure 1a illustrates the diffraction pattern of the precursor. This measurement is important and essential in demonstrating later that all the ZrO_2_ has reacted.

From the Figure 1a it can be seen that the ZrO_2_ contains two crystalline phases. The first main crystalline phase is monoclinic, marked by the red squares. The peaks correspond well to the standard PDF data No: 00-037-1484. The second phase detected shows presence of tetragonal lattice structure (JCPDS 01-079-1769), marked with the blue squares. By comparing the intensities of these two crystalline phases, we can conclude that the monoclinic phase is the major component in the solid mixture and the calculated ratio for the monoclinic to tetragonal phases is 8:1. The XRD pattern of the synthesized Zr sample after acid treatment is shown in Figure 1b. The diffraction peaks of zirconium are marked with black diamonds. Comparison of the diffraction peaks in Figure 1a and Figure 1b indicates that the product does not contain residues of the precursor, ZrO_2_. For example, the peak at 2θ = 28.23° in Figure 1a, which is the most intense ZrO_2_ diffraction peak, is not observed in Figure 1b, or is buried in the background. We cannot rule out however, the existence of Zr_x_O_y_ in the background. Even if such impurities exist their amount is less than 5%. The absence of MgO-peaks confirms that it is completely removed by the acid treatment. Although the XRD pattern of the synthesized Zr sample is very similar to the X-ray pattern of PDF No: 01-089-3045 for metallic Zr, the peak positions are shifted slightly to lower angles as compared with those reported in the PDF. This shift can be explained either by considering that the lattice is highly distorted due to the high temperature of the reaction, or due to defects in the lattice caused by the elimination of the oxygen. Another explanation could be that Mg^+2^ ions have not been fully removed and are located in the lattice, and these slightly enlarge its size. Moreover, it should be noted that the wide background for 2θ between 20° and 24° in Figure 1b, is due to the mylar layer that covers the sample in the specially inert XRD cell [12] designed to avoid the oxidation of the Zr sample. However, further proof for the successful fabrication of metallic zirconium is required.

**Figure 2:**
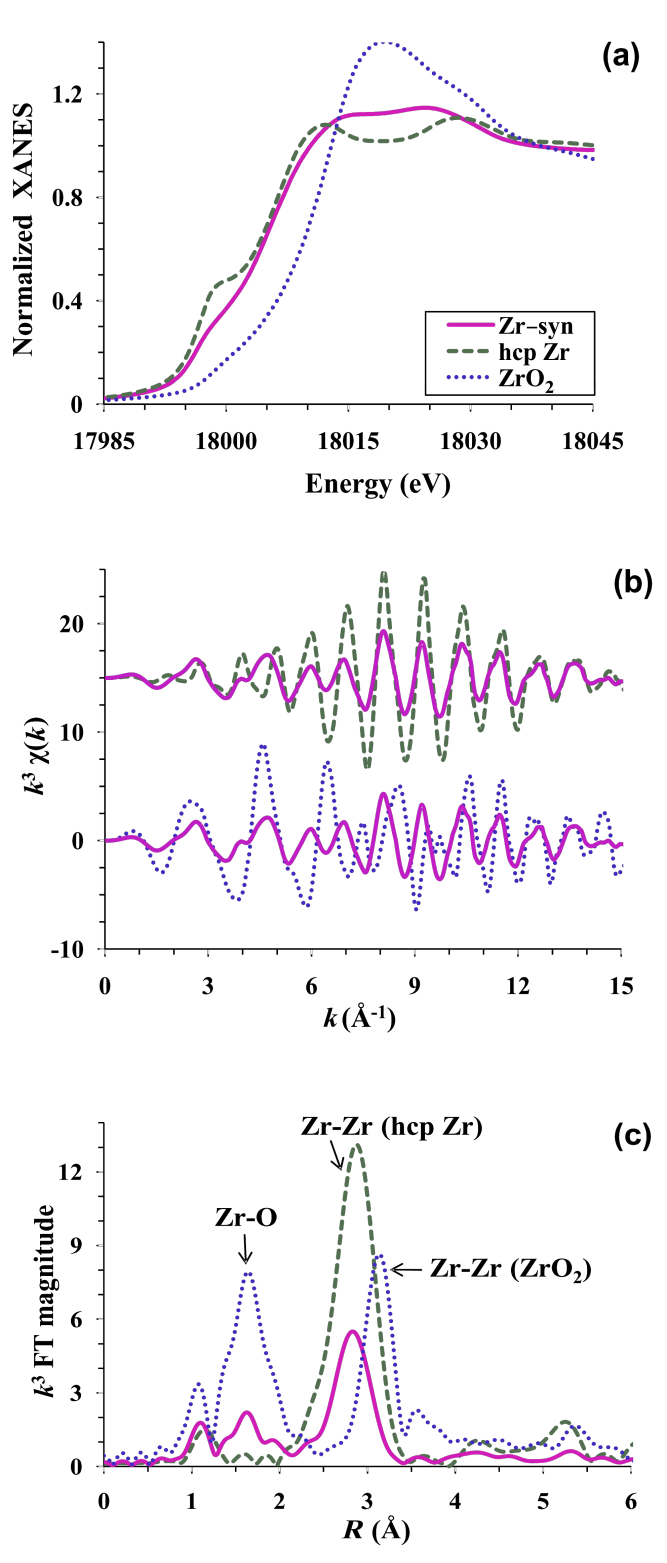
Zr K-edge (a) normalized XANES, (b) *k*^3^-weighted EXAFS and (c) corresponding Fourier Transform of Zr-syn sample (solid line), standard ZrO_2_ (dotted line) and metallic hcp Zr (dashed line).

X-ray absorption spectroscopy (XAS) was employed to confirm the formation of metallic Zr synthesized (Zr-syn) by RAPET of ZrO_2_. The techniques of XAS, namely X-ray absorption near edge structure (XANES) and extended X-ray absorption fine structure (EXAFS), are used to probe local structural details [13] around specific metal atoms and to discern the oxidation states of the metal, here the zirconium atom. The Zr K-edge XAS of Zr-syn is compared with the standards, ZrO_2_ (also used as a starting material) and metallic hexagonal close packed (hcp) Zr as shown in Figure 2. The normalized XANES spectrum (Figure 2a) shows a comparison of the starting ZrO_2_ material and Zr-syn with the metallic hcp Zr standard. The general appearance of the XANES of starting material is very similar to the spectra reported [14] by Li et al. for the monoclinic polymorph of ZrO_2_. This observation confirms that the monoclinic phase is the major component in the starting material. The presence of small amounts (a few percent) of other polymorphs cannot be discounted, as XAS is an averaging technique. This finding is consistent with XRD data where the monoclinic polymorph was identified as the major phase. The main K-edge position of Zr-syn sample is at a much lower energy than that of ZrO_2_ and at a slightly higher energy than the hcp Zr standard. This observation reveals that the majority of Zr in Zr-syn has been reduced to the metallic Zr^0^ oxidaton state. An analysis of the XANES of the Zr-syn sample using a linear combination fit to Zr and ZrO_2_ standards indicates that in the Zr-syn sample at least 75% of the Zr atoms are in the fully reduced Zr^0^ state.

Figure 2b represents the normalized *k*^3^-weighted EXAFS spectra of Zr-syn, compared separately with hcp Zr (top) and ZrO_2_ (bottom). The corresponding Fourier transforms (FT), which represents a pseudo radial distribution function of the Zr probe atoms is shown in Figure 2c. The FT is uncorrected for photoelectron phase-shifts, so the peaks occur ~0.3–0.4 Å lower than the actual distances. The contribution from the first Zr–O and Zr–Zr correlations in ZrO_2_ and the first Zr–Zr correlation in the hcp Zr standard is marked. Note that the Zr–Zr correlation distance in metallic hcp Zr is much smaller than that found in ZrO_2_. The EXAFS (Figure 2b) of Zr-syn is distinctly different for low *k*-values (below ~5 Å^−1^) when compared to hcp Zr, but for high *k*-values (above ~5 Å^−1^) the spectra of Zr-syn shows a better similarity to hcp Zr metal. Oxygen contributes at low *k*-values, indicating the presence of some oxygen coordination in the Zr-syn sample. The presence of this Zr-O contribution is evident in the FT of the Zr-syn sample, which shows some intensity in the region between 1.2−2.1 Å. In monoclinic ZrO_2_, each zirconium atom is surrounded by a distorted shell of 7 oxygen atoms [15] at an average distance of ~2.16 Å: This contributes to the first peak in the FT of ZrO_2_. Assuming that the Zr-O environment is similar to that present in the starting material, we estimate (by using the FT peak intensity in the 1.2–2.1 Å range and approximately scaling both the magnitude and the real part of the FT) that ~25% of the Zr atoms in Zr-syn have a first shell oxygen environment similar to that of ZrO_2_. This is consistent with the XANES determination. The second peak in FT centered at ~3.2 Å in ZrO_2_ and at ~2.8 Å in the hcp Zr standard represents the metal–metal interaction. The Zr–Zr contribution in Zr-syn sample is at same position as in the standard hcp Zr, which unequivocally confirms the presence of a strong metallic Zr component. The reduced amplitude in the Zr-syn as compared to hcp Zr could result either from the formation of under coordinated nanosize particles and/or because of some cancellation effect by out of phase EXAFS contributions from the oxidized portion of Zr. A detailed analysis of this is beyond the scope of this short paper. Together the XANES and EXAFS confirm the formation of metallic Zr as a major component in Zr-syn sample along with presence of some oxidized Zr. Thus, the formation of a significant fraction of metallic Zr by the RAPET method is confirmed by XAS characterization.

**Figure 3:**
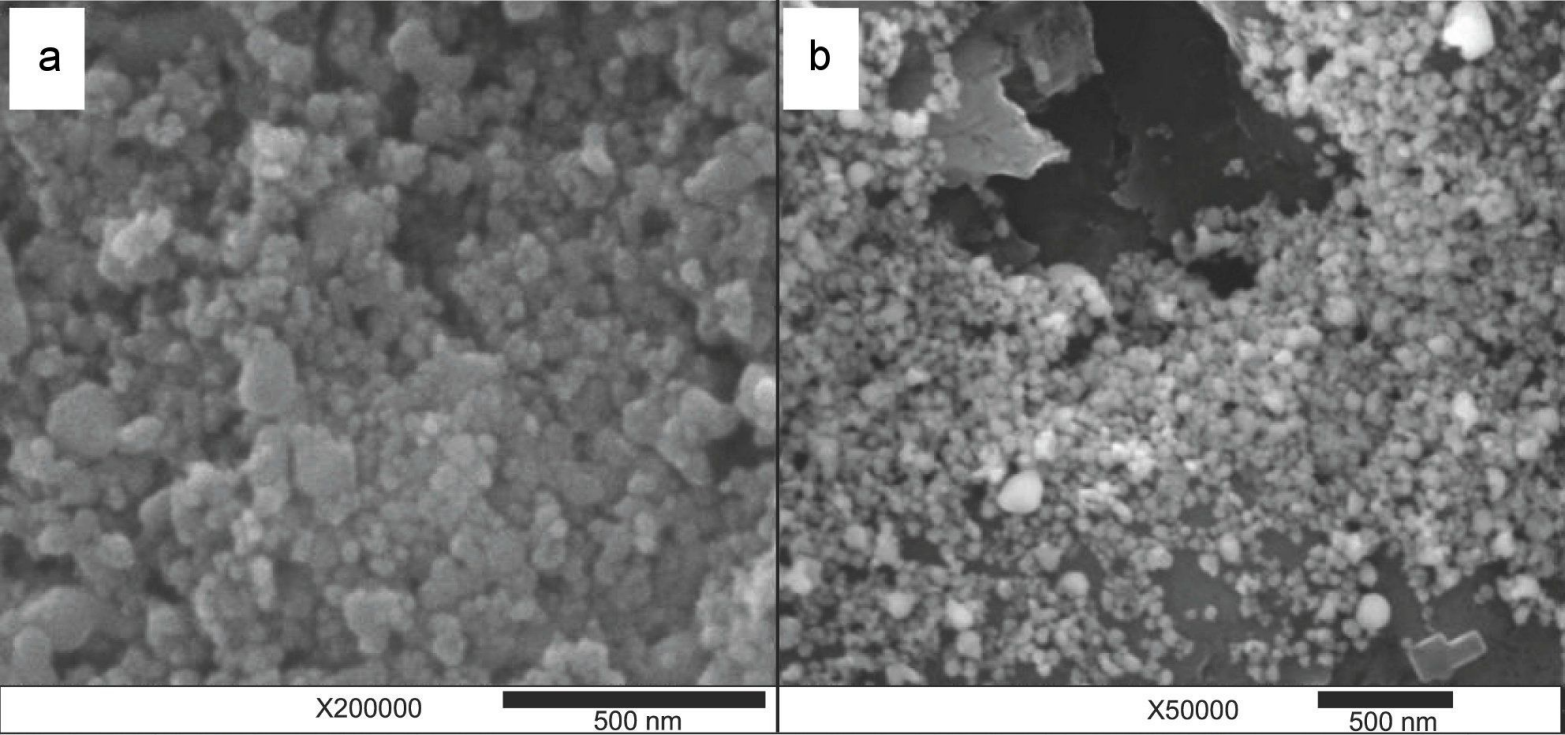
SEM images for (a) ZrO_2_ and (b) Zr.

**Figure 4:**
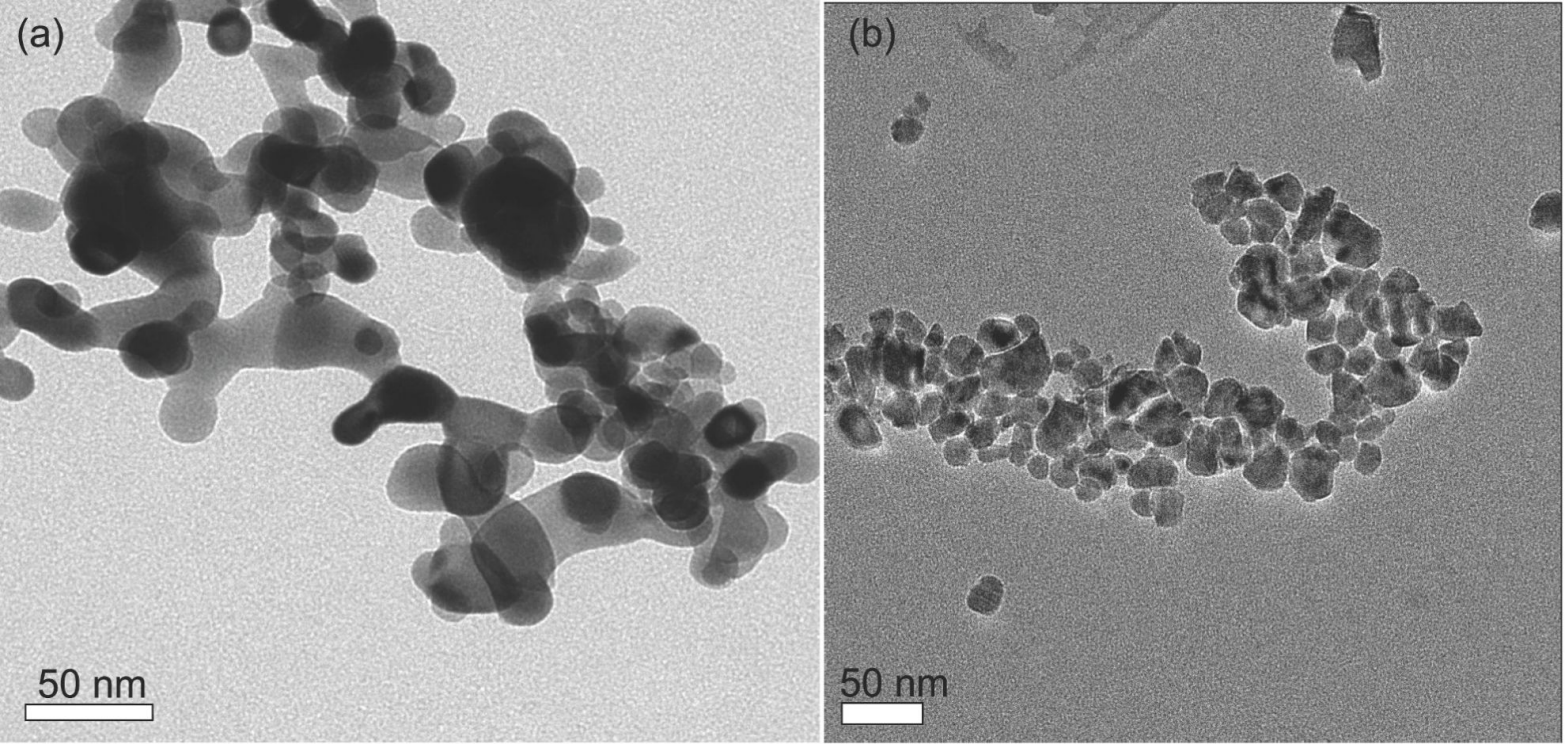
(a) TEM images of ZrO_2_, the reactant (b) HRTEM of the reaction product.

Further characterization measurements were conducted for defining the morphology of the product. It should be mentioned that a minimal exposure of the obtained product to air, caused the powder to burn. In any case the oxygen level, as measured by elemental analysis, was low even in cases in which we had to expose the sample for a short period to air. Figure 3a depicts the SEM image of the ZrO_2_, precursor. The morphology of the zirconium dioxide consisted mostly of spheres. The average size of the zirconium oxide nanoparticales was measured by using the scion image program averaged over 100 particles. The average size of the ZrO_2_ particles was found to be 55 ± 5 nm. However, it seems that even these particles are aggregates of smaller, 10–20 nm, particles. The same process was repeated for the synthesized Zr nanoparticles and their size determined from Figure 3b by the scion program. Figure 3b depicts the morphology of the product. The average size of metallic Zr was found to be around 63 ± 6 nm. These numbers show that there is no change in the particle size during the reaction that the zirconia undergoes. In addition, TEM measurements were carried out on the precursor (Figure 4a) and the product (Figure 4b). While it is clear that agglomeration takes place as a result of nanoparticle interaction, the size of individual particles can be discerned and their sizes correspond well with the SEM values. The particles detected in Figure 4b are different from each other, although some particles have sizes similar to those measured from the SEM picture, ~55 nm. In addition, EDAX measurements were performed on the HCl-treated product. These measurements show the presence of 87.9% Zr and 12.2% O (atomic percentages). We did not find any traces of Mg, which indicates that the HCl has completely removed the MgO.

## Conclusion

This paper reports a simple and rapid method for the synthesis of metallic zirconium nanoparticles with an average size of 63 nm by reducing zirconia at 750 °C. The TEM images show particles without a specific shape and the XRD measurements indicate that the product was metallic Zr without any traces of the by-product, MgO. Distinct peaks of the starting material, ZrO_2_, are not observed in the XRD pattern. In addition, XANES measurements found that in the Zr sample at least 75% of the Zr atoms are in the fully reduced Zr^0^ state.

## Experimental

Chemicals, ZrO_2_, and Mg, were obtained from Aldrich. A 3 mL closed vessel cell was assembled from stainless steel (made by HAM-LET, Israel). A 3/8” union part was plugged from both sides by standard caps. For the synthesis, 0.500 g of zirconium dioxide (ZrO_2_) and 0.200 g of magnesium powder (molar ration 1:2, respectively) were introduced into the cell, and the cell was closed tightly at room temperature under a nitrogen atmosphere (in a nitrogen-filled glovebox). The cell (Let-Lok^®^) was placed inside an iron pipe at the center of the tube furnace. The temperature was raised at a rate of 10 °C per minute. The closed cell was heated at 750 °C for 5 h. The reaction proceeded under the autogenic pressure of the precursors. The closed vessel cell (Let-Lok^®^) was gradually cooled (5 h) to room temperature. Since the Zr nanoparticles are highly reactive, special care is required for avoiding their oxidation. The cell was opened inside the glovebox and collected material (mixture of metallic Zr and MgO) was treated with HCl for 2 h in order to dissolve the byproduct, MgO and then centrifuged (Hettich Universal 32) at 9000 rpm for 20 min. The separated solid material was collected and washed twice with water, once with ethanol, and then dried under vacuum.

It is important to emphasize that the obtained product must be kept under an inert atmosphere, such as in a glovebox, otherwise even a minimal exposure to air will cause the sample to ignite.

X-ray diffraction (XRD) patterns were collected using a Bruker AXS Advance powder X-ray diffractometer (Cu K_α_ radiation, λ = 1.5418 Å). The XRD of synthesized sample was measured inside the special XRD cell designed [12] to avoid the reaction of air sensitive samples with atmospheric oxygen.

For XAS measurements, about 12 mg each of ZrO_2_ (Aldrich) and Zr-syn, were homogeneously mixed with 110 mg of pre-dried boron nitride (BN, Sigma Aldrich) and pelletized in a 13 mm diameter die under a pressure of 5000 psi. The Zr-syn pellet was made inside a glovebox under an inert atmosphere. This pellet was inserted in a thin aluminized mylar pouch and sealed with Kapton^®^ tape. Further XAS measurement of Zr-syn sample was carried out in a holder with a constant flow of He gas to minimize exposure to the ambient atmosphere. Zr K-edge XAS measurements of the aforementioned sample and ZrO_2_ standard were performed in transmission mode at Sector 20 bending magnet (20-BM) beam line of the Advanced Photon Source at Argonne National Laboratory, USA. Reference spectra of standard elemental Zr metallic foil (hcp Zr) was also recorded simultaneously for internal energy calibration. The first inflection point of hcp Zr was defined at 17995.88 eV [16]. Subsequent, XAS data reduction followed standard procedures using Athena software [17].

Transmission electron microscopy measurements were carried out with a transmission electron microscopy (TEM) instrument FEI Tecnai™ Spirit 120 kV bioTWIN. Samples for TEM were prepared by ultrasonically dispersing the products into absolute ethanol, placing a drop of this suspension onto a copper grid coated with an amorphous carbon film or onto a copper plate, and then drying in vacuum. High resolution TEM (HRTEM) was measured using a JEOL JEM-2100 electron microscope. The morphology and the size of the nanoparticles were studied by scanning electron microscopy (SEM) with a JEOL-JSN 7000F instrument. Oxygen elemental analysis was measured by CHNS-O analyzer Thermo Flash EA 1112 series.
